# Cancer du sein de l'homme: à propos de 6 cas

**DOI:** 10.11604/pamj.2013.16.70.2345

**Published:** 2013-10-28

**Authors:** Kamilia Laabadi, Sofia Jayi, Fatimazohra Fdili Alaoui, Hakima Bouguern, Hikmat Chaara, My Abdelilah Melhouf, Karim Ibn Majdoub Hassani, Said Ait Laalim, Hicham Anoun, Imane Toughrai, Khalid Mazaz

**Affiliations:** 1 Service de gynéco-obstétrique 2, CHU Hassan II, Fes, Maroc; 2 Service de chirurgie générale, CHU Hassan II, FES, Maroc

**Keywords:** Cancer du sein chez l'homme, diagnostic, traitement, facteurs pronostiques, Breast cancer in men, diagnosis, treatment, prognostic factors

## Abstract

Le but de ce travail était d'analyser les caractéristiques cliniques, histologiques, thérapeutiques et pronostiques du cancer du sein chez l'homme. Il s'agissait d'une étude rétrospective portant sur six patients colligés au service de gynécologie obstétrique II, CHU Hassan II durant la période 2009-2012. L’âge moyen de nos patients est de 65.3 ans. Il s'agit dans 83.3% des cas, d'une tumeur rétroaréolaire dont la taille moyenne est de 44.16 mm. Nous avons retrouvé 4 (66.7%) T4, 1 (16.7%) T3 et dans un cas, une tumeur inclassable. Le type histologique le plus représenté est le carcinome canalaire infiltrant (66.7%). Le taux d'envahissement ganglionnaire axillaire est de 66.7%. L'hormonodépendance de ces tumeurs est prouvée dans 100% des cas. La survie à cinq ans est en cours d’évaluation. L'envahissement ganglionnaire, l'invasion du derme, le stade clinique TNM sont des facteurs qui influencent significativement la survenue de métastases. Aucun de ces facteurs de risque n'est apparu significatif en termes de survie globale. Le cancer du sein chez l'homme est une maladie rare (environ 1% des cancers du sein) au pronostic sombre. Le diagnostic est le plus souvent tardif et les lésions sont traitées à des stades avancés.

## Introduction

Le cancer du sein, première pathologie maligne chez la femme, reste une maladie rare chez l'homme. Et bien que de nombreuses études fassent référence à cette pathologie, elles restent rares et sont dans la majorité des cas des études rétrospectives avec un nombre limité de patients. C'est une pathologie méconnue du grand public et la découverte d'un nodule mammaire chez un homme ne suscite pas la même inquiétude que chez la femme. Ainsi, le diagnostic est le plus souvent retardé rendant le pronostic plus sombre. La prise en charge thérapeutique a été extrapolée depuis des acquis chez la femme avec certaines particularités pour le traitement chirurgical. A travers notre travail nous envisageons d'insister sur les caractéristiques épidémiologiques, diagnostiques, thérapeutiques et pronostiques de cette entité rare.

## Patients ét observations

### Cas 1

Il s'agit d'un Patient de 70 ans, sans antécédents pathologiques notables, présentant un nodule mammaire gauche, augmentant progressivement de volume depuis 1an, sans autres signes associés. L'examen clinique trouve une tumeur rétro mamelonnaire gauche mesurant 3cm de diamètre, de consistance ferme, mal limité, mobile par rapport aux deux plans associé à une adénopathie axillaire gauche indolore et fixe par rapport au plan superficiel, classée T3N2bMx. La mammographie trouve une opacité rétro mamelonnaire gauche de 35 mm, grossièrement arrondie, de contours irréguliers, contenant des micro calcifications d'allure suspect (figure A). Le complément échographique confirme la présence d'une lésion tissulaire en rétro mamelonnaire gauche de contours irréguliers hétérogène associée à des adénopathies axillaires et une sus claviculaire suspectes. Cette lésion est classée ACR V. La biopsie du nodule est en faveur d'un carcinome canalaire infiltrant de grade III de SBR, 5MSBR. L’étude immunohistochimique objective que 80% des cellules tumorales expriment les récepteurs à l’‘strogène et que 30% expriment les récepteurs à la progestérone. Le bilan d'extension objective la présence d'une métastase pulmonaire. Une chimio-radiothérapie est indiquée mais le patient est perdu de vue.

### Cas 2

Un patient âgé de 59 ans, sans antécédents particuliers, consulte pour une tuméfaction rétro mamelonaire gauche indolore évoluant depuis un an. L'examen clinique objective une masse rétro aréolaire de 5cm de grand axe indolore, de consistance ferme, fixe par rapport au plan superficiel avec une adénopathie homolatérale de 1cm. Cette tumeur est classée T4bN1Mx. la mammographie, trouve une opacité rétro mamelonaire du sein gauche mal limitée de contours irréguliers avec des microcalcifications classées ACR V. L’échographie mammaire a objectivé une lésion tissulaire du sein gauche classé ACR V avec des adénopathies homolatérales suspectes. Dans un premier temps, le patient a bénéficié d'une biopsie chirurgicale. A l'histologie, il s'agit d'un carcinome canalaire infiltrant grade II de SBR (5MSBR), avec 70% des cellules tumorales exprimant les récepteurs à l’‘strogène alors que les récepteurs à la progestérone sont négatifs. La recherche de HER2 n'a pas été faite par manque de moyen. Le bilan d'extension est sans particularité. Devant le caractère localement avancé, le patient a reçu 6 cures de chimiothérapie à base d'AC 60 puis On a complété par un Haishted. Le résultat histologique est en faveur de carcinome canalaire infiltrant grade III de SBR (5MSBR) avec envahissement du derme. L'analyse histologique du muscle grand pectoral n'a pas objectivé d'envahissement. Le curage ganglionnaire a ramené 25 ganglions dont 4 sont métastatiques. Le patient a bénéficié d'une radiothérapie puis fut mis par la suite sous hormonothérapie à base de tamoxiféne.

### Cas 3

Un patient âgé de 68 ans, diabétique sous insulinothérapie depuis 4ans, tabagique chronique pendant 10 ans, sevré il y 5 ans, et qui présente depuis 5 ans une tuméfaction mammaire droite associée à des mastodynies et des signes inflammatoires en regard. L'examen clinique initial a objectivé une masse rétroaréolaire faisant 7/4 cm classée T4d N1 MO PEV 1. La mammographie est techniquement impossible à réaliser puisque la lésion du sein droit est incompressible. L’échographie mammaire a objectivé une lésion tissulaire rétroareolaire faisant 6.4/ 3.4 cm classée ACR 5. La microbiopsie est en faveur de carcinome apocrine du sein gauche, grade II de SBR, 3MSBR. 90% des cellules tumorales expriment les récepteurs oestrogéniques avec une intensité de 3+. 90% des cellules tumorales expriment les récepteurs progéstéroniques avec une intensité de 3+. Le bilan d'extension est sans particularité. Le patient a reçue une chimiothérapie néo-adjuvante à base de 6 cures d'AC 60, suivie d'un Haischted. L'examen histologique définitif a conclu à un carcinome canalaire infiltrant de 4cm de grade II de SBR, 3MSBR, infiltrant le muscle en profondeur et le derme. On note également la présence d'une composante intracanalaire extensive sans emboles vasculaires, avec présence de la maladie de Paget du mamelon. Le curage ganglionnaire a ramené 2 ganglions positifs sur 24 sans effraction capsulaire ni emboles vasculaires. la tumeur est classée p T4 N1Mx, Chevalier: grade 4, Sataloff: grade T-D pour la tumeur et N-D pour les ganglions. La réponse thérapeutique est estimée à moins de 50%. Le patient est adressé pour une radiothérapie. Une hormonothérapie a été indiquée.

### Cas 4

Un patient de 56 ans, tabagique chronique depuis 36 ans et qui présente depuis 1 an un nodule lenticulaire du sein gauche augmentant progressivement de volume avec notion d'ulcération en regard. L'examen clinique a objectivé une lésion périaréolaire gauche de 5.5/5 cm ulcérée classée cliniquement T4d N0 MO. Vu l'aspect trop ulcérée de la lésion mammaire gauche ne permettant pas une étude échographique adéquate. Il n'y a pas d'adénopathie axillaire. La biopsie chirurgicale a été faite et a trouvé un CCI de grade I de SBR (2MSBR), pas de carcinome canalaire in situ ni d'emboles vasculaires. RE: 60% (+), RP: 50% (+), HER2: score 1 (négatif). Bilan d'extension est sans anomalie. Le patient a reçu 3 cures de chimiothérapie néo adjuvante à base d'AC60 avec une nette amélioration clinique. Un Hashteid a été réalisé dont le résultat anatomopathologique est en faveur d'un carcinome canalaire infiltrant de 1,5 cm de grade I de SBR (2MSBR) sans composante intracanalaire ni emboles vasculaires le curage ganglionnaire a ramené 12 ganglions négatifs. Le HER 2 négatif. La réponse thérapeutique est estimée à 90%. Le patient est readressé en oncologie pour complément de chimiothérapie et une hormonothérapie par la suite.

### Cas 5

Patient de 79 ans présentant depuis 2ans un nodule du sein gauche augmentant progressivement de volume associé à des mastodynies. Le patient a bénéficié dans une autre formation d'une échomammographie objectivant une lésion rétroaréolaire de 4cm classée ACR V puis a bénéficié d'une tumerectomie en privé et dont l’étude anatomopathologique est en faveur d'un CCI grade II de SBR avec des limites de résection saines. L’évolution a été marquée par une récidive au lit tumoral pour lequel il a bénéficié d'une 2ème tumerectomie revenant en faveur de CCI grade II de SBR avec contingent intra-canalaire (5% du volume tumoral) de grade intermédiaire et limites non saines, Puis il nous a été référé pour prise en charge. L'examen clinique a objectivé une induration périmamelonnaire gauche sur la cicatrice de tumerectomie. On a opté pour un patey et résultat anatomopathologique est revenu en faveur d'un carcinome micropapillaire du sein de 1cm de grand axe, grade II de SBR, 3MSBR, absence de composante in situ, absence d'emboles vasculaire, absence de maladie de Paget du mamelon. L'immunohistochimie a révélé des RE: positifs à 90%, des RP positifs à 90%, le HER2: négatif et le Ki 67: 20%. Le curage ganglionnaire a ramené un ganglion positif sur 13 ganglions avec effraction capsulaire. La tumeur est classée p T1N1Mx. Une chimiothérapie et radiothérapie ont été indiquées ainsi qu'une hormonothérapie.

### Cas 6

Patient de 60 ans, sans ATCDs pathologiques notables, admis pour prise en charge d'un nodule du sein droit évoluant depuis 2 ans et chez qui l'examen clinique objective un nodule de 2/2 cm, rétroareolaire droit classé cliniquement T4bN1MO. L’échomammographie: présence d'une lésion droite de la jonction des quadrants internes classée ACR V avec des adénopathies axillaires droites suspectes. La microbiopsie du nodule est en faveur d'un carcinome colloïde muqueux associé à une petite composante canalaire classique de grade I de SBR (2MSBR, pas vu d'emboles vasculaires. Un haschteid modifié a été réalisé et dont le résultat histologique est en faveur d'un carcinome mucineux avec une composante de carcinome canalaire infiltrant, de grade I de SBR, 2MSBR, mesurant 3.4 cm, absence de carcinome in situ. Le curage ganglionnaire a ramené 37 ganglions dont 28 sont métastatiques avec emboles vasculaires et rupture capsulaire. La tumeur est classée p T2N3Mx. L’étude des récepteurs oestrogénique montre un marquage de 100%, l’étude des récepteurs progestéroniques montre un marquage de 90% alors que l'HER2 est négatif et le Ki 67 est à 30%. Une chimiothérapie a été démarrée et on prévoit une radiothérapie et une hormonothérapie.

## Discussion

Le cancer du sein chez l'homme est une maladie rare. Sa prévalence est d'un pour 100000 individus et il représente moins de 1% des cancers du sein et moins de 1% de l'ensemble des néoplasies masculines [1, 2]. L'incidence de cette pathologie semble relativement stable en Europe [1], mais Giordano et al montrent une augmentation de 26% entre 1973 et 1998 aux Etats Unis [3]. L’âge moyen lors du diagnostic varie entre 63 et 71 ans [4] ce qui correspond à cinq ans de plus par rapport à l’âge de découverte du cancer du sein chez la femme avec des extrêmes allant de 5 à 93 ans [1, 3, 4]. Sa distribution est unimodale. Dans notre série l’âge dépasse 50 ans avec des extrêmes de 56 et 79 ans. L'étiopathogénie du cancer du sein chez l'homme reste obscure, cependant de nombreux facteurs de risques semblent pouvoir être incriminés. L´obésité par aromatisation des androgènes et la cirrhose éthylique par l´élévation de sex steroid binding globulin provoquent un état d′hyperoestrogènie [5]. Les anomalies testiculaires comme l'ectopie testiculaire, l'orchite, l'orchidectomie, les hernies inguinales congénitales et la stérilité sont des facteurs associés à un risque élevé de cancer du sein. Les seins ombiliqués, les antécédents de traumatisme mammaire et la transsexualité (incluant la castration chirurgicale et chimique) semblent impliqués [5–7]. L'hypogonadisme, présent dans le syndrome de Klinefelter, fait de lui un facteur de risque classiquement admis (risque relatif de 20 à 50 fois par rapport à un homme sans anomalies génétiques) mais cette prédisposition serait expliquée aussi par l'apport exogène de la testostérone, transformée en ‘strogène dans le tissu adipeux périphérique [5].

Les hommes ayant une histoire familiale de cancer du sein féminin ont 2,5 fois plus de risque de développer un cancer du sein. L´exposition aux champs électromagnétiques affecte l´activité de la glande pinéale avec diminution du taux de mélatonine (hormone à action antioestrogénique) [4, 8]. Les antécédents d′irradiation (période de latence de 20 à 30 ans) et l´exposition aux hautes températures [5] ont été aussi incriminés et enfin, une consommation régulière d'alcool a été aussi retrouvée [5, 9, 10]. En revanche la gynécomastie idiopathique ou d'origine médicamenteuse n'est pas incriminé dans la survenue de cette pathologie [4, 5, 11].

### Génétique

Le cancer du sein chez l´homme est augmenté en cas d´antécédents familiaux comme c'est le cas chez la femme [5]. En général, pour J.R. Weiss [5], une histoire familiale de cancer du sein chez un homme ou une femme au premier degré est associée à un risque multiplié par deux à trois. Les mutations des gènes *BRCA1 et BRCA2*sont incriminées dans une proportion du cancer du sein chez l´homme mais avec un moindre risque absolu que chez la femme et avec une fréquence plus faible [12, 13].

Les mutations du *BRCA1* ne sont présentes que chez 10 à 16% des patients sans antécédents familiaux [12, 14, 15]. Les mutations du BRCA2 sont plus fréquentes dans le cancer du sein chez l´homme et sont estimées de 4 à 16% [16]. C´est en raison de la prévalence de ces mutations dans le cancer du sein chez l´homme qu´un conseil génétique doit être proposé. Le risque cumulatif de cancer du sein chez l´homme est de 6.3% à 70 ans [7]. Le pourcentage d´expression de la mutation du BRCA2 en cas de cancer du sein chez l´homme est variable d´une population à une autre (3.6 à 40%) ce qui reflète les différences génétiques entre les populations [17, 18]. Le syndrome de Cowden est une maladie autosomique dominante caractérisée par le développement d´hamartomes multiples associés à la mutation germinale du gène PTEN suppresseur de tumeur, localisé en 10q23. Il est responsable de cancer du sein chez l´homme et chez la femme [5, 19]. La mutation de CHEK2 qui est une kinase jouant un rôle dans la réparation de l´ADN CHEK2*1100delC a été incriminée dans le développement de cancer du sein chez l´homme [5]. L´âge moyen de développement d´un cancer du sein chez un homme ayant un syndrome de Klinefelter est de 58 ans. Trois à quatre pour cent des cancers du sein chez l´homme sont associés à un syndrome de Klinefelter. Dans notre série, aucun patient n´avait d´histoire familiale de cancer et par conséquent aucun n´a eu d´enquête génétique.

### Diagnostic

Le cancer du sein chez l´homme se présente, dans la plupart des cas, sous la forme d´une tuméfaction douloureuse subaréolaire, d´une rétraction mamelonaire ou d´un écoulement sanglant [5, 20, 21]. Lorsque ce nodule survient chez un homme âgé et en surcharge pondérale, son diagnostic sera encore plus difficile. Comme chez la femme, il existe une légère prépondérance d´atteinte à gauche. Le diagnostic différentiel est la gynécomastie, qui touche 30% des hommes [22]. Dans notre série, tous nos patients ont consulté pour un nodule rétro mamelonaire. La mammographie permet de différencier une gynécomastie d´un cancer invasif mammaire qui se manifeste par un nodule excentrique irrégulier à bords spéculés [23, 24]. La sensibilité et la spécificité de la mammographie dans le diagnostic du cancer du sein chez l´homme sont de 92 et 90% respectivement [23]. L´échographie mammaire complète la mammographie et fournit des informations sur l´état ganglionnaire. L´imagerie est systématiquement complétée par une microbiopsie. Nos patients ont bénéficié d'une biopsie chirurgicale dans 3 cas (patients gérés initialement dans une autre formation), d'une microbiopsie dans 2 cas et d'une tumorectomie à titre externe dans un cas.

### Histologie

Le carcinome canalaire in situ (CCIS) représente 10% des cancers du sein chez l´homme [3]. La plupart du temps, il s´agit du type papillaire et cribriforme de bas grade [25]. Le carcinome lobulaire in situ est rare étant donné l´absence de lobules terminaux mais a été décrit en association avec le carcinome lobulaire invasif dans le cancer du sein chez l´homme [19]. Dans notre série, il n´a pas été décrit de carcinome lobulaire in situ sur les pièces d´exérèse alors que le carcinome canalaire in situ a été retrouvé chez un seul patient. En ce qui concerne les carcinomes invasifs, les types histologiques sont similaires pour les deux sexes mais les distributions relatives différentes [3]. Les données résultant de la surveillance de 2000 patients dans le SEER (Surveillance, Epidemiology, and End Results cancer registry) ont montré que 93.7% des cancers du sein chez l´homme sont canalaires ou non classés, 2.6% sont papillaires, 1.8% est mucineux et seulement 1.5% sont lobulaires [3]. Dans notre série le carcinome canalaire est le prédominant représentant 83.3% des cas.

Il existe une forte hormonodépendance de ces tumeurs. Approximativement, 90% expriment les récepteurs aux œstrogènes et 81%, les récepteurs à la progestérone [3]. Dans notre série, les récepteurs hormonaux étaient fortement positifs dans tous les cas. Le taux d´expression des récepteurs hormonaux dans le cancer du sein chez l´homme est significativement plus important que chez la femme et augmente, comme chez la femme, avec l´âge du patient [3, 26]. Une étude récente sur une série de 75 patients a montré que 5% des cancers du sein chez l´homme ont une surexpression en her2-neu [27]. Le rôle des récepteurs aux androgènes n´est pas clair et n´a pas de conséquences sur le pronostic du cancer du sein chez l´homme [28, 29].

Le bilan d´extension comprend le marqueur tumoral CA15.3 qui a comme intérêt surtout la surveillance, une radiographie du thorax, une échographie abdominale et une scintigraphie osseuse, (non réalisée seulement chez un patient par manque de moyen). Le stade de la tumeur est déterminé par l´utilisation de la classification de l´American Joint Committee on Cancer (AJCC), qui tient compte de la taille tumorale, de l´atteinte ganglionnaire et des métastases à distance [30].

### Pronostic

Le cancer du sein chez l'homme semble avoir un pronostic plus péjoratif que chez la femme. La taille tumorale ainsi que l´atteinte ganglionnaire sont deux facteurs pronostiques importants dans le cancer du sein chez l´homme [3]. Les hommes ayant une tumeur de 2 à 5 cm ont un risque de décès majoré de 40% par rapport à ceux dont la tumeur mesure moins de 2 cm de diamètre maximum. Nous n´avons pas trouvé, dans la littérature, de précisions sur les stades de découverte de cancer du sein chez l´homme comparé au stade de diagnostic chez la femme. En revanche, au même stade, la survie était la même. En cas d´atteinte ganglionnaire, il y a un risque supplémentaire de 50% de décès qu´en cas de ganglions indemnes de métastases. En analyse univariée, la négativité des récepteurs hormonaux et le grade tumoral sont associés à un mauvais pronostic de survie [3, 31]. Le cancer du sein chez l´homme dû à une mutation du BRCA2 survient plus tôt et avec un pronostic plus sombre. En général, le pronostic pour les patientes et les patients avec un cancer du sein est similaire [3]. Les taux de survie chez les hommes sont plus faibles, mais cela est dû au fait que le diagnostic est posé à un stade plus avancé de la maladie [3].

### Traitement

Au stade précoce: Le traitement local est le même que chez la femme. La plupart des hommes est traitée par une mastectomie radicale modifiée associée à un curage axillaire ou à la lymphadénectomie sélective [32]. Historiquement, c´est la chirurgie radicale qui est le plus souvent pratiquée bien que des études rétrospectives aient démontré que le pronostic était le même en cas de chirurgie moins invasive [33]. Dans notre série, la mastectomie avec curage axillaire et résection du muscle grand pectoral était indiquée d'emblé chez seulement 2 patients vu que les autres patients ont consulté à un stade avancé. La tumerectomie semble donner un moins bon contrôle local de la maladie. Dans une série de 31 cas de carcinome canalaire in situ, Cutuli et al. montrent trois rechutes après six tumerectomies (50%) alors qu'ils ne retrouvent qu'un seul cas de rechute pour 25 mastectomies (4%). La petite taille de la glande mammaire rend difficile le passage en marges saines. La tumorectomie n'est donc pas recommandée [1]. Donc, le traitement chirurgical conservateur n'a pas sa place dans le traitement du cancer du sein chez l'homme, d'une part, du fait du faible volume mammaire et, d'autre part, de l'acceptation aisée de la mastectomie. En revanche, tous les autres traitements, chirurgicaux (curage axillaire ou ganglion sentinelle), radiothérapie, chimiothérapie, hormonothérapie (tamoxifène ou anti-aromatases) et biothérapie (transtuzumab) peuvent faire partie de l'arsenal thérapeutique. Le curage axillaire reste nécessaire [11]. Le curage ganglionnaire axillaire est compliqué de lymphoedéme du membre supérieur invalidant dans 10 à 25% des cas. La lymphadénectomie sélective a été récemment évaluée dans le cancer du sein chez l´homme [34, 35]. Étant donné la rareté de cette maladie, la sensibilité et la spécificité du ganglion sentinelle n´ont pas été évaluées mais plusieurs séries ont été publiées établissant la faisabilité de la technique dans cette indication [36–40]. La radiothérapie est plus souvent indiquée chez l´homme après mastectomie que chez la femme en raison de la fréquence d´atteinte mamelonnaire ou cutanée [32]. La radiothérapie évite une récidive locorégionale mais les études n´ont pas démontré de différence en terme de survie [32]. Dans notre série, l´indication de radiothérapie a été retenue chez 5 de nos patients. Il existe peu d´informations concernant l´efficacité de la chimiothérapie en adjuvant en cas de cancer du sein chez l´homme. Une seule étude prospective a été publiée dans ce but chez 24 hommes ayant bénéficié d´une chimiothérapie par CMF (cyclophosphamide, méthotrexate, fluoro-uracile) avec un taux de survie de plus de 80% à cinq ans, et significativement plus important que dans une cohorte similaire [41]. Des séries rétrospectives ont montré la diminution du risque de récurrence chez les patients [11]. Ce sont souvent les mêmes protocoles de chimiothérapie qui sont utilisés pour la femme. Dans le centre de l´université de Texas M.D.-Anderson Cancer [42], la chimiothérapie est indiquée si la taille tumorale est supérieure à 1 cm et en cas d´atteinte ganglionnaire. Les anthracyclines sont proposées seules si les ganglions sont indemnes et en association avec les taxanes en cas d´atteinte ganglionnaire. L'hormonothérapie est indiquée si les récepteurs sont présents [4, 30]. Les études rétrospectives ayant évalué le tamoxifène en adjuvant ont montré la diminution du risque de récidive et de décès. La toxicité du tamoxifène chez l´homme n´a pas été étudiée. Certaines études ont décrit une intolérance au produit, des thromboses veineuses, une diminution de la libido, des troubles de l´humeur et des bouffées de chaleur [43]. En ce qui concerne les antiaromatases, une seule série a été publiée sur cinq patients métastatiques mais sans réponse objective [44]. Deux cas ont été rapportés récemment concernant des patients traités par le létrozole avec une diminution significative de la masse tumorale [45, 46] mais d´autres investigations sont nécessaires afin de déterminer l´efficacité des antiaromatases chez l´homme. [Àgrave] ce jour, les données sont insuffisantes pour recommander les antiaromatases en adjuvant chez l´homme.

Au stade métastatique: L´attitude thérapeutique est la même que chez la femme. L'hormonothérapie est souvent indiquée étant donné la positivité fréquente des récepteurs. Farrow et Adair [47] ont décrit le cas d´un cancer du sein chez l´homme ayant régressé après orchidectomie. Historiquement, l´orchidectomie, la surrénalectomie et l´hypophysectomie ont été pratiquées afin de contrôler le cancer du sein métastatique mais sont remplacées actuellement par l'hormonothérapie. Le tamoxifène est la molécule de choix avec un taux de réponse de 50% [48]. Les agonistes de la LH-RH ont également été utilisés avec ou sans les antiandrogènes et ont prouvé leur efficacité dans le cancer du sein chez l´homme [49]. La chimiothérapie trouve sa place chez les patients ayant des récepteurs hormonaux négatifs ou bien en cas de résistance à une hormonothérapie de première ligne. La chimiothérapie palliative en cas de progression rapide de la maladie peut être indiquée. L´efficacité du trastuzumab en cas de surexpression chez l´homme n´est pas prouvée mais serait à tenter chez les hommes métastatiques ayant une surexpression en HER-2 pour Volm et al. [50].

Cancers primitifs ou secondaires: Les hommes ayant survécu au cancer du sein ont un risque élevé de développer un deuxième cancer primitif comme un cancer controlatéral primitif du sein [34] avec un risque absolu de 1.75%. Auvinen et al. [35] estiment que les hommes avec antécédent de cancer du sein ont 30 fois plus de risque de développer un cancer du sein controlatéral. Pour les autres localisations, tel le mélanome ou le cancer de la prostate, le risque est élevé chez les survivants, surtout en cas de mutation génétique.

## Conclusion

Le cancer du sein chez l'homme est une maladie rare. Les facteurs de risque sont multiples et variés Le diagnostic se fait à un âge plus tardif que chez la femme et à un stade plus avancé. La présentation clinique de la maladie diffère un peu chez l'homme. Toutes les variétés histologiques peuvent se voir chez l'homme avec une rareté du carcinome lobulaire. Les récepteurs hormonaux sont souvent positifs avec une rareté de surexpression en her2-neu chez l'homme. La méthode du ganglion sentinelle est techniquement possible. L'hormonothérapie joue un rôle important dans le traitement et le tamoxifène reste la molécule du choix. Le pronostic du cancer du sein chez l'homme est le même que chez la femme au même stade. Des études multicentriques sont nécessaires afin d'optimiser la prise en charge de patients atteints de cette maladie.
